# Diagnostic and therapeutic recommendations for the treatment of hyperphenylalaninemia in patients 0–4 years of age

**DOI:** 10.1186/s13023-018-0911-6

**Published:** 2018-09-29

**Authors:** Ania C. Muntau, Marcel du Moulin, Francois Feillet

**Affiliations:** 1 University Children’s Hospital, University Medical Center Hamburg-Eppendorf, Hamburg, Germany; 2 Department of Pediatrics, Hôpital d’Enfants Brabois, CHU Nancy, Vandoeuvre les Nancy, France

**Keywords:** Infants, Diagnosis, Therapy recommendations, Sapropterin dihydrochloride, Phenylketonuria, BH_4_

## Abstract

**Background:**

Treatment of phenylketonuria (PKU) with sapropterin dihydrochloride in responsive patients from an early age can have many advantages for the patient over dietary restriction alone. Accordingly, approval of sapropterin in the European Union was extended in 2015 to include patients aged 0–4 years, bringing the treatment age range in line with that in the USA and providing an additional treatment option for those patients with PKU who are responsive or partially responsive to treatment with sapropterin. Subsequently, European guidelines have been published on the diagnosis and management of patients with PKU. However, testing for PKU can be demanding and requires particular expertise. We have compiled experience-based, real-world guidance in an algorithmic format to complement the published guidelines, with the overall aim to achieve optimized and individualized care for patients with PKU.

**Results:**

Our guidance covers aspects such as how to perform, monitor and interpret appropriate biochemical measures to achieve effective patient management and desired outcomes, how to perform a tetrahydrobiopterin (BH_4_) loading test to assess responsiveness in newborns, and how to initiate sapropterin treatment in patients from birth. We also provide our expert opinion on starting pharmacotherapy in patients who were previously managed by diet alone.

**Conclusions:**

Real-world-based guidance is particularly important in managing therapeutic strategies in newborns with PKU to achieve optimal long-term outcomes and will serve as a complement to the other published guidelines.

**Electronic supplementary material:**

The online version of this article (10.1186/s13023-018-0911-6) contains supplementary material, which is available to authorized users.

## Introduction

Phenylketonuria (PKU) is a rare autosomal-recessive metabolic disorder in which a deficiency in the hepatic enzyme phenylalanine hydroxylase (PAH) results in complete or partial inability to metabolize phenylalanine (Phe) into tyrosine [1]. This can lead to elevated blood Phe concentrations which, if left untreated, can cross the blood–brain barrier and cause many detrimental effects, including profound mental retardation, seizures and autistic behavior [2]. The overall incidence of PKU varies widely around the world, from as high as 1 in 2600 births in Turkey [3] to fewer than 1 in 100,000 births in Japan [4, 5]; other reported incidences are 1 in 4500 births in Ireland [6, 7], 1 in 10,000 births in the United Kingdom [7, 8], 1 in 11,000 births in China [9, 10] and 1 in 15,000 births in the United States [11].

Screening for PKU in newborns enables early diagnosis and therapeutic intervention to prevent the most severe consequences of the disorder. The current standard therapy is adherence to appropriate treatment (including a Phe-restricted diet) for life to maintain blood Phe concentrations within recommended ranges to achieve the best clinical outcomes. While diet remains the cornerstone of treatment, in a subset of patients with partial PAH deficiency, use of tetrahydrobiopterin (BH_4_, an essential cofactor of PAH) alone or in addition to diet has been shown to further lower elevated blood Phe levels [12–14]. Sapropterin dihydrochloride (Kuvan^®^, BioMarin, CA, USA), a synthetic formulation of the 6R-isomer of BH_4_ that activates residual PAH enzyme activity in patients of all ages with BH_4_ responsive PKU, has been commercially available for several years. This agent was approved for the treatment of hyperphenylalaninemia (HPA) in adult and pediatric patients with PKU in 2007 in the USA and in 2008 in Europe. This treatment was initially authorized without age restriction in the USA but only in patients older than 4 years in Europe [15]. The effectiveness of BH_4_ treatment has been demonstrated in a number of short-term [16–19] and long-term studies [18, 20–24], which have included patients aged 0–4 years and shown the benefit of treatment in this age range. The advantages of initiating BH_4_ therapy at an early age include increasing natural protein intake during a critical time of growth and development and increasing the likelihood of adherence to treatment [22]. Accordingly, in 2015, approval of sapropterin was extended in Europe to children aged 0–4 years [15, 22, 25].

The adverse events reported in children treated with sapropterin were essentially the same as those reported in adults. In clinical trials of children aged 4 years and above, adverse events with an incidence of ≥10% were headache and rhinorrhea; those occurring with an incidence of ≥1% to < 10% were pharyngolaryngeal pain, nasal congestion, cough, diarrhea, vomiting, abdominal pain and hypophenylalaninemia [15]. In children aged below 4 years who received treatment with sapropterin (10 or 20 mg/kg/day), the most commonly reported adverse reactions were hypophenylalaninemia, vomiting and rhinitis [22]. Hypersensitivity reactions, including serious allergic reactions and rash, were also reported.

Because only a subset of patients with PKU responds to treatment with BH_4_, it is important to detect BH_4_ responsiveness at the neonatal stage, to determine the appropriate course of management: those who are fully responsive will be treated only with sapropterin, those who are partially responsive will be treated with sapropterin and Phe-restricted diet, and those who are not responsive will be treated with Phe-restricted diet only. In 2009, the European working group on PKU described a protocol for optimizing sapropterin use in PKU management [26]. In 2014, the American College of Medical Genetics and Genomics (ACMG) and Genetic Metabolic Dietician’s International (GMDI) released new guidelines for the optimal treatment of PKU that included guidance on sapropterin pharmacotherapy, stipulating that BH_4_ responsiveness should be investigated by performing a BH_4_ loading test in all PKU patients [6, 27], except those who have two null mutations in the *trans* position and, therefore, have no chance of being BH_4_ responsive [28]. European guidelines for the diagnosis and management of patients with PKU were published recently [29]. Because sapropterin was not authorized in Europe for patients younger than 4 years of age, there is little experience of performing the neonatal loading test and implementing BH_4_ treatment after a positive neonatal screening test result; in some regions, there is provision for the patient to receive sapropterin after a positive screening test, but this is subject to local regulations and will vary from country to country. To address this and to complement the published guidelines, our manuscript provides practical insights on performing a BH_4_ loading test in newborns and the possibility of initiating BH_4_ therapy from birth, based on treatment algorithms. These recommendations are designed to complement those set out in the published guidelines, aiming to facilitate a personalized treatment management plan for patients with PKU.

### Diagnosis of HPA in newborns

**Fig. 1:**
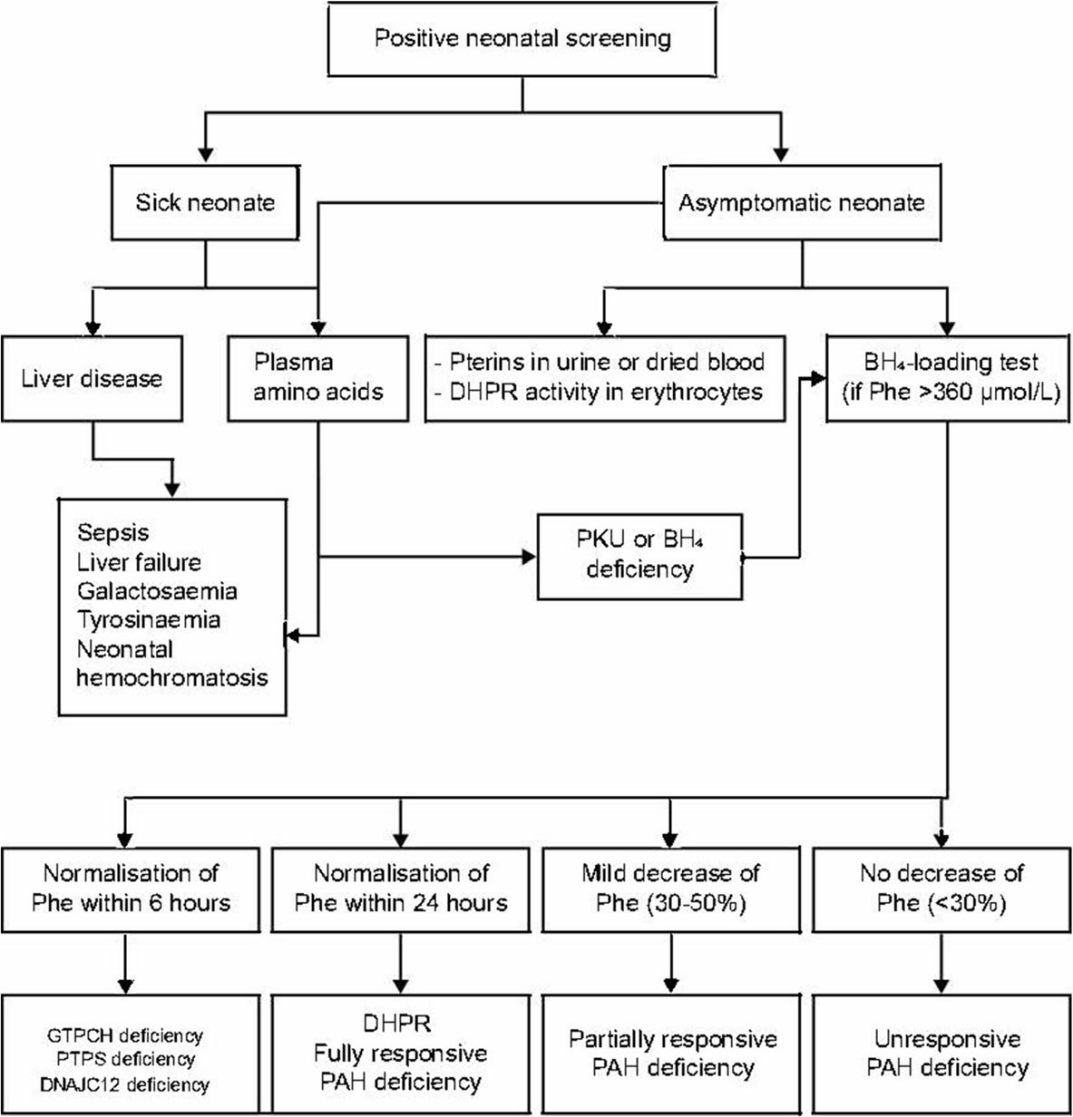
Diagnosis algorithm of different types of PKU. BH_4_: Tetrahydrobiopterin; DHPR: Dihydropteridine reductase; GTPCH: Guanosine triphosphate cyclohydrolase; PAH: Phenylalanine hydroxylase; Phe: Phenylalanine; PKU: Phenylketonuria; PTPS: 6-pyruvoyl-tetrahydropterin synthase. We recommend that a full panel of investigations is conducted, even in patients with unknown genotype who do not respond in the BH_4_ loading test. This will ensure that the subtype of PKU can be identified, which may be beneficial for the ongoing management of the patient

Following a positive test for HPA in neonatal screening using tandem mass spectroscopy, the elevated Phe concentration (> 120 μmol/L) and increased Phe/tyrosine ratio (> 3 if tandem mass spectrometry is used) should be confirmed using a second blood sample [29]. All causes of neonatal HPA must be ruled out, including liver disease, PAH deficiency, genetic defects in the synthesis or regeneration of BH_4_ or *DNAJC12.* Further investigations and genotyping should also be performed in the neonatal period, to assess the probability of BH_4_ responsiveness and hence guide treatment options (Fig. 1) [28]. As stated earlier, patients with two null mutations in the *trans* position will not respond to treatment with BH_4_.

#### Performing the BH_4_ loading test in newborns

There are several benefits to performing the BH_4_ loading test in newborns (Table 1). The test must be performed as early as possible following the positive test for HPA. Ideally, this should be once it has been determined that the neonate does not present with liver disease or general sickness and if blood Phe concentrations reach or exceed the level of 360 μmol/L. The neonate must be on a normal diet during the test.

**Table 1:** Justification and benefits of the neonatal BH_4_ loading test

	Criteria/benefit of the neonatal BH_4_ loading test
1	BH_4_ treatment is now available from birth in Europe (as well as USA)
2	The Phe concentration in the newborn with a genetic defect in phenylalanine metabolism is spontaneously evaluated
3	The neonatal test allows the early diagnosis of GTPCH or PTPS deficiency
4	The normalization of blood Phe concentration will be reached sooner in the responsive neonates
5	The 24-h test only delays the management of the condition by 24 h and allows treatment to begin within the first 10 days of life
6	A complete phenotype of the patient is generated (Phe level and BH_4_ responsiveness)
7	The parents are rapidly informed of the types of treatment that are available for their child
8	Some patients will be able to avoid the dietetic treatment from birth
9	The good safety profile of this molecule is well established
10	The neonatal test avoids performing a Phe load in young patients after the newborn period

*BH*_*4*_ Tetrahydrobiopterin, *GTPCH* Guanosine triphosphate cyclohydrolase I, *Phe* Phenylalanine, *PTPS* 6-pyruvoyl-tetrahydropterin synthase

In the test, a baseline capillary blood sample is collected to measure the Phe level followed by administration of sapropterin 20 mg/kg (one dose) dissolved in breast milk or infant formula (Additional file 1). Following sapropterin administration and throughout the entire test, a normal feeding regimen (e.g., breast milk or regular infant formula) must be provided on demand and Phe-free medical formula must not be given. Capillary blood samples to measure Phe levels are taken at 4, 6, 8, 12, 16 and 24 h after sapropterin administration (Additional file 2). The test must be done over a period of 24 h because it is not possible to delay treatment in neonates with classical PKU, who will not respond to BH_4_, for longer than 24 h. The results must be available within 24 h after the end of the test, and treatment can then be implemented; this can be sapropterin alone if the Phe level decreases to within the target range (fully responsive patients) or a Phe-restricted diet (with or without sapropterin) if the blood Phe concentrations remain above the target range.

There are some data to suggest that a negative BH_4_ test may not rule out long-term responsiveness to BH_4_; therefore, except in patients with two null mutations, there may be a case for retesting for BH_4_ responsiveness when the child is older [30].

#### Interpreting the results of the BH_4_ loading test

**Fig. 2:**
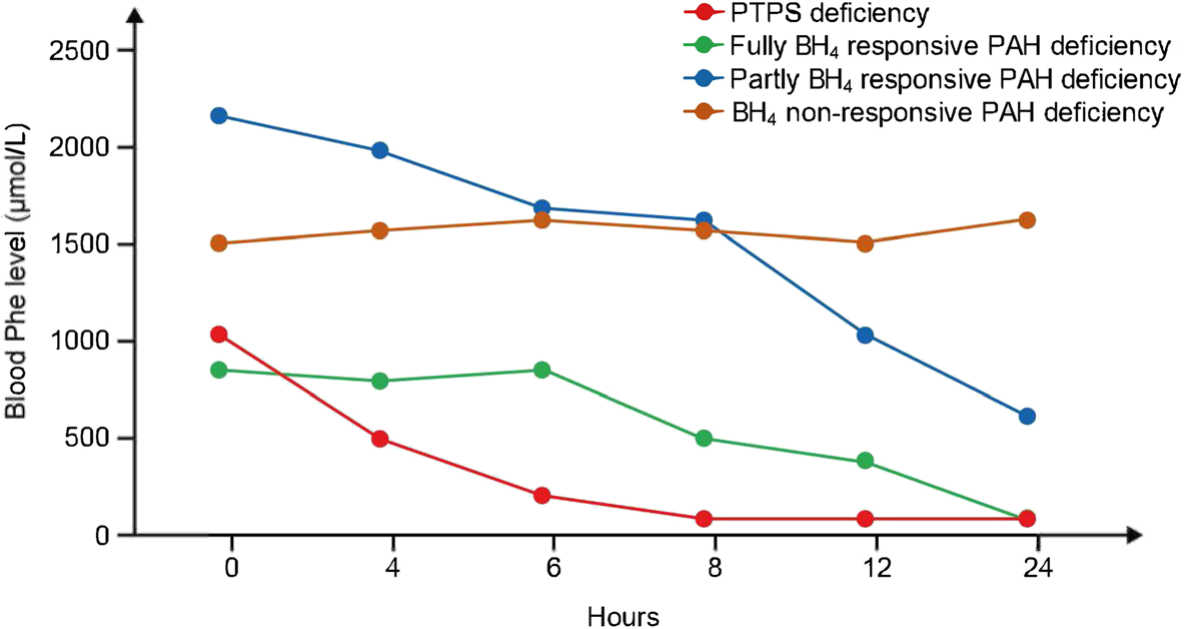
Illustrative examples of neonatal BH_4_ loading test outcomes. BH_4_: Tetrahydrobiopterin; PAH: Phenylalanine hydroxylase; Phe: Phenylalanine; PTPS: 6-pyruvoyl-tetrahydropterin synthase

**Fig. 3:**
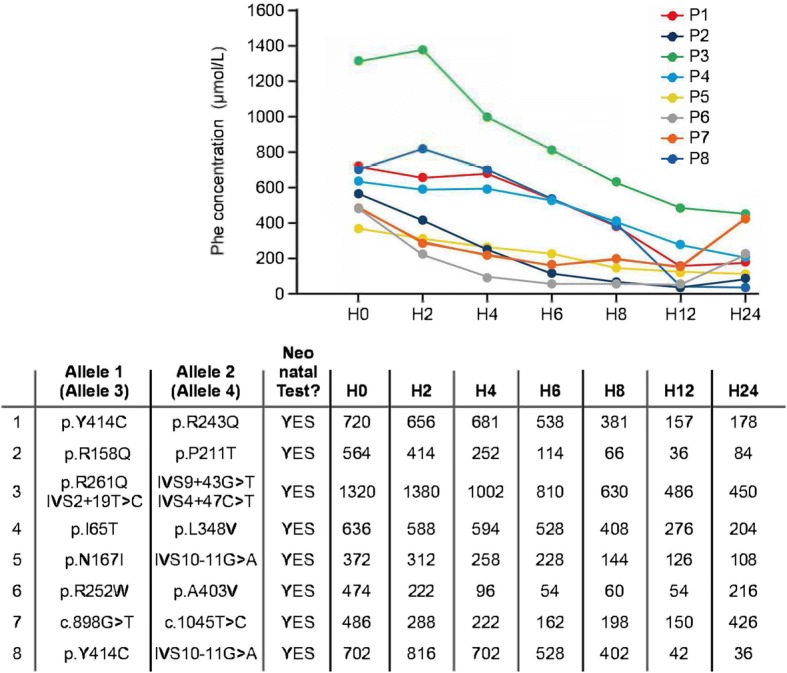
Case studies to show response during the test phase in eight BH_4_-responsive patients. BH_4_: Tetrahydrobiopterin; Phe: Phenylalanine. P1–8, patients 1–8

The possible different responses in blood Phe concentration that can be observed following sapropterin administration are illustrated in Fig. 2. A decrease in blood Phe concentration of ≥30% at any point during the test indicates the presence of BH_4_ responsive HPA. A rapid fall in the Phe concentration to a value approaching the target range (120–360 μmol/L) within 2–6 h suggests a genetic disorder in the synthesis of BH_4_ (known as ‘primary BH_4_ deficiency’: guanosine triphosphate cyclohydrolase [GTPCH] or 6-pyruvoyl-tetrahydropterin synthase [PTPS] deficiency) (Fig. 2). If the blood Phe concentration decreases to within the target range in 24 h, this suggests dihydropteridine reductase (DHPR) deficiency or fully responsive PAH deficiency (Fig. 2). In this outcome, the result of the pterin (neopterin or biopterin) analysis from urine or dried blood and blood DHPR activity in erythrocytes should be retrieved. If the diagnosis of a genetic disorder of the synthesis or regeneration of BH_4_ is confirmed, then additional management steps must be initiated (such as the determination of cerebrospinal fluid neurotransmitter levels, serum prolactin and further genetic testing). Further guidance on the diagnosis and management of ‘primary BH_4_ deficiency’ can be found elsewhere [31]. If genetic testing for PAH and biopterin deficiencies is normal, *DNAJC12* deficiency should be considered. The results of BH_4_ loading tests in eight patients with BH_4_ responsive PAH deficiency with different genotypes are shown in Fig. 3.

It is worth noting that the threshold of ≥30% is arbitrary; therefore, patients with a smaller decrease in Phe concentration can still show a clinically meaningful increase in Phe tolerance during a long-term therapy trial, mainly if the BH_4_ loading test has been performed while blood Phe concentrations are low. Conversely, patients showing a reduction in blood Phe concentration of ≥30% might not respond satisfactorily to sapropterin during a long-term therapy trial. This is often the case if the reduction is only slightly greater than 30% or is only observed at one test time point. Hence, a positive BH_4_ loading test result should be critically reviewed and confirmed by a long-term therapy trial with a meaningful benefit determined in terms of Phe tolerance. In patients showing a decrease of blood Phe of more than 30%, a 1-month trial can be performed. This can be done with sapropterin alone if the Phe level dropped to within the target range (fully responsive patients) or sapropterin combined with a Phe-restricted diet if the blood Phe levels remain above the target range. Unresponsive patients should be started on a Phe-restricted diet.

### Sapropterin treatment for newborns with BH_4_ responsive PAH deficiency

Two potential scenarios could arise after completion of the BH_4_ loading test. If it is possible to obtain the results the day after the loading test is performed, BH_4_ treatment can be implemented immediately (in the case of a full response). If it is not possible to obtain the results in this timeframe, dietetic treatment must begin, either with only Phe-free medical food if the baseline Phe value > 600 μmol/L or a mixed diet of breast milk/complete infant formula can be administered if baseline Phe value < 600 μmol/L until the results of the test are obtained; in such cases, there is a high likelihood of BH_4_ responsiveness. If the results indicate BH_4_ responsive PAH deficiency, pharmacotherapy with sapropterin can begin.

For fully responsive neonates, sapropterin treatment can be implemented. The starting dose usually given in Europe is 10 mg/kg/day, although many physicians prefer to use a starting dose of 20 mg/kg; the use of either dose is acceptable but some patients may have a better response to lower doses of sapropterin than they do to higher doses, depending on their genotypes [32]. Sapropterin is administered in the morning as a single dose dissolved according to the guidance for water dilution, which is the diluent specified in the package insert (Additional file 1). However, the rate and extent of absorption of sapropterin is influenced by food. The absorption of sapropterin is higher after a high-fat, high-calorie meal compared with during the fasted state, which results, on average, in 40–85% higher maximum blood concentrations achieved over 4–5 h after administration [15, 33]. For example, milk is a fat-containing food that is much more effective than water or fruit juice for the optimal absorption of sapropterin. After administration of sapropterin, blood Phe concentrations must be monitored tightly (daily if possible). We recommend that parents must be educated from an early stage to take fasting capillary blood samples in the morning. Daily monitoring of blood Phe concentration allows for calculation of mean blood Phe concentrations and, on this basis, for gradual (weekly or fortnightly) titration of the Phe intake to the target range by adherence to the following algorithm:

- Mean blood Phe concentration ~ 360 μmol/L: do not change Phe intake.
- Mean blood Phe concentration < 360 μmol/L: increase Phe intake by 20% or, if the child is already on a normal diet, decrease the dose of sapropterin by 2 mg/kg/week or do not increase the sapropterin dose in response to the child gaining weight.
- Mean blood Phe concentration > 360 μmol/L: reduce Phe intake by 10–30%, depending on the degree of elevation of the blood value [21, 22, 34].

It can take several weeks to discern the maximum Phe intake that can be tolerated while keeping blood Phe concentrations in the target range. The above algorithm should, therefore, be used for at least 4 weeks. If a starting dose of 10 mg/kg/day was chosen and the effect on dietary Phe tolerance was not satisfactory, an increase in the dose to 20 mg/kg/day should be considered. After the patient has reached maximum normal Phe intake, with blood Phe concentrations lower than 360 μmol/L, a critical evaluation of the benefit of treatment with sapropterin should be carried out. Consideration of the responses to certain questions may be helpful in this evaluation (Table 2).

**Table 2:** Questions for the evaluation of sapropterin treatment

• Is the Phe tolerance higher than expected based on the baseline blood Phe concentrations and the genetic test results? • How much natural protein can the patient consume without blood Phe levels reaching above 360 μmol/L? • Does the patient require a Phe-free amino acid mixture to cover the recommended age-appropriate protein intake? • Is the patient tolerating the medication?	

**Fig. 4:**
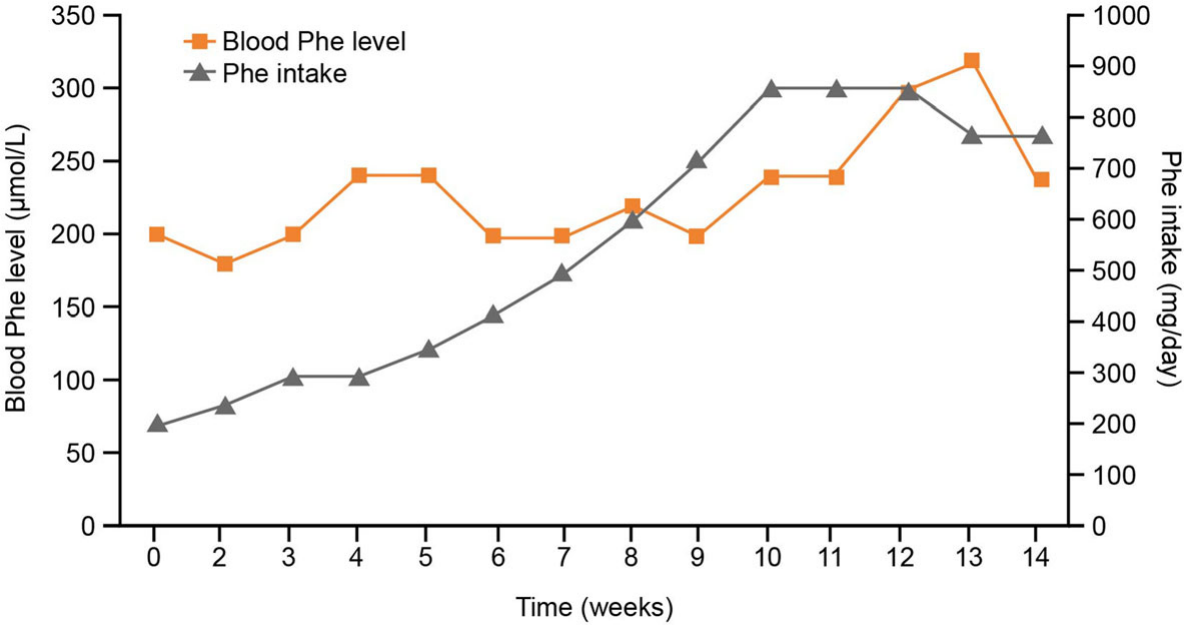
Diagrammatic representation of the course of a successful therapy trial with sapropterin in a newborn with BH_4_-responsive PAH deficiency. BH_4_: Tetrahydrobiopterin; PAH: Phenylalanine hydroxylase; Phe: Phenylalanine; PKU: Phenylketonuria

The course of a successful therapy trial with sapropterin in a newborn with BH_4_ responsive deficiency is shown in Fig. 4.

### Sapropterin treatment for a child younger than 4 years with prior dietary management

**Fig. 5:**
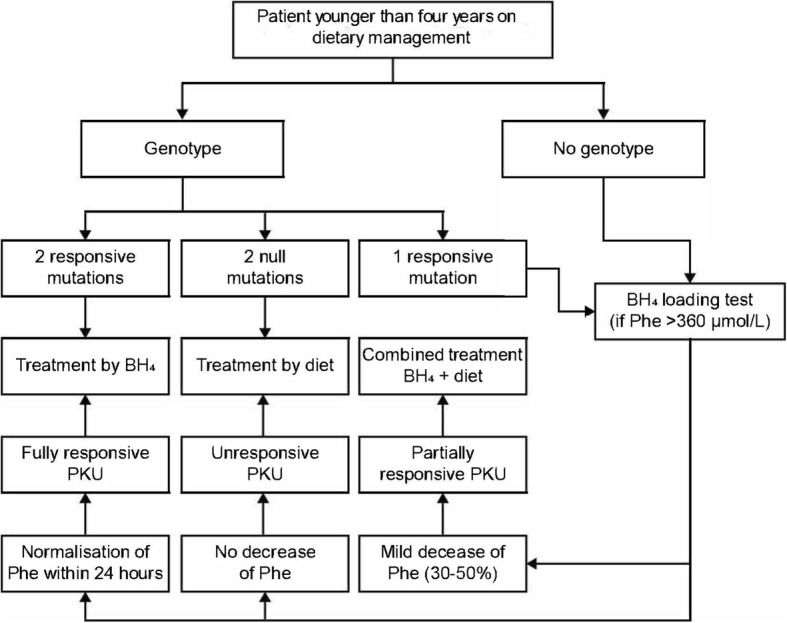
Algorithm of BH_4_ loading test for children after neonatal period and aged < 4 years. The BH_4_ loading test must be performed in relation to the genotype (the presence of only one responsive mutation or of one mutation of unknown responsiveness). When the genotype is unknown, the BH_4_ loading test should be performed, if available. BH_4_: Tetrahydrobiopterin; Phe: Phenylalanine; PKU: Phenylketonuria

When considering pharmacotherapy for a child younger than 4 years of age with prior dietary management, the treatment algorithm in Fig. 5 together with the following questions are useful (Table 3).

**Table 3:** Questions to consider before pharmacotherapy for a child <4 years

• Has a BH_4_ loading test already been performed over 24 h (in the neonatal period) or over 48 h (in children older than neonates) and did the test indicate that the child was BH_4_ responsive? • Does the patient have a genotype that is associated with a good probability of being BH_4_ responsive?	

If the answers to either of the questions are positive, further tests for BH_4_ responsiveness should be performed. It should be noted that prior to approval of sapropterin in children aged 0–4 years, in most countries in Europe it was possible to authorize the BH_4_ loading test but not the treatment; hence, it was possible to have the situation of known positive test without having treated the child. If the results of a BH_4_ loading test are not available, genotyping may help to estimate the probability of BH_4_ responsiveness (Table 4).

**Table 4:** Responses according to whether or not the genotype is known

• Two null mutations *in trans*: no reason to perform further tests on the responsiveness to BH_4_; treat with Phe-restricted diet. • Two responsive mutations: treat the patient with sapropterin. • One responsive mutation or unknown genotype: consider further tests for BH_4_ responsiveness. • If the genotype is not identified, further tests of BH_4_ responsiveness should be considered.	

It should be noted that it is not easy to perform a BH_4_ loading test in patients who are well managed by diet and thus have low blood Phe concentrations (and this indirectly supports performing the test during the neonatal period when the Phe level is naturally elevated). In these cases, a treatment trial with sapropterin dihydrochloride is recommended instead. If you are not able to perform a treatment trial, a BH_4_ loading test should be performed. In this case, the Phe concentration at the beginning of the test should be stable at > 360 μmol/L, which generally requires an increase in Phe intake. This often causes difficulties and needs to be done under close management.

#### Performing a sapropterin therapy trial in a patient younger than 4 years previously managed by diet alone

Therapy trials are divided into screening and test phases (Table 5) and an algorithm is used to adjust the dietary Phe intake.

**Table 5:** Summary of steps involved in a sapropterin therapy trial

Screening Phase	
• Diet alone • Food diary • Determination of baseline Phe tolerance (PheTol_0_) • Determination of the baseline quantity of protein from the amino-acid mixture (amino-acid supplement [AAS_0_]) • Determination of the baseline blood Phe concentration (Phe_0_)	
Test Phase	
• Sapropterin 10–20 mg/kg per day • Food diary • Determination of the Phe tolerance on therapy (PheTol_1_) • Determination of the quantity of protein from the amino-acid mixture on therapy (AAS_1_) • Determination of the Phe concentration on therapy (Phe_1_) • Continuing the test phase based on the algorithm until steady state is reached	
Evaluation	
• Calculate the differences: ­ PheTol_x_ – PheTol_0_ ­ AAS_x_ – AAS_0_ ­ Phe_x_ – Phe_0_ • Evaluation and critical benefit analysis in the team	

##### Screening phase

The patient should be free of any infections or illness at the start of the test. The diet is initially continued as usual, and a food diary is kept for 1 week. From this, baseline Phe tolerance (PheTol_0_) and baseline quantity of protein from the amino acid mixture (amino acid supplement [AAS_0_]) are calculated. Daily fasting blood samples are collected in the first week, and the mean blood Phe concentration (Phe_0_) is determined. The duration of this phase may be longer than 1 week in some cases.

##### Test phase

Sapropterin is administered in the morning at 10 mg/kg/day as a single dose dissolved in milk (Additional file 1). A food diary is again kept for 1 week, and the current Phe tolerance (PheTol_1_) and quantity of protein from the amino-acid mixture (AAS_1_) are calculated. Daily fasting blood samples are collected during the week and the mean blood Phe concentration (Phe_1_) is determined.

##### Confirmation of BH_4_ responsiveness by the Phe intake algorithm

The algorithm below defines how to adjust the Phe intake based on the blood Phe concentrations.

- Mean blood Phe concentration ~ 360 μmol/L: do not change Phe intake.
- Mean blood Phe concentration < 360 μmol/L: increase Phe intake by 20% or, if the child is already on a normal diet, decrease the dose of sapropterin by 2 mg/kg/week or do not increase the sapropterin dose in response to the child gaining weight.
- Mean blood Phe concentration > 360 μmol/L: reduce Phe intake by 10–30%, depending on the degree of elevation of the blood value [21, 22, 34].

After this, daily fasting blood samples continue to be taken and daily blood Phe concentrations (Phe_2_ to Phe_x_) are determined. We would like to emphasize that a high number of daily blood samples is helpful to judge metabolic control; however, if this is not attainable, the frequency of blood sampling can be reduced.

##### Duration of the test phase

One month after starting the trial, the quantity of amino acid mixture administered (AAS_1_) is adjusted. If there is a further increase in Phe intake, and thus in the intake of natural protein, other adjustments are made at a later time (AAS_x_). The algorithm loop is repeated until steady state is reached (with any further increase in Phe intake, the blood Phe concentration is above 360 μmol/L in two successive samples). The Phe intake is then initially reduced by 10%. If blood Phe concentrations in the subsequent week remain outside the target range, Phe intake is reduced by a further 10% (illustrated in Fig. 4). If the effect on dietary Phe tolerance was not satisfactory at a dose of 10 mg/kg/day, an increase in the dose to 20 mg/kg/day should be considered. At the end of the test phase, the following values are documented: difference between Phe_0_ and Phe_x_; difference between PheTol_0_ and PheTol_x_; and difference between AAS_0_ and AAS_X_.

Regular blood monitoring and dietary and nutritional assessments are recommended for patients during long-term treatment with sapropterin [29].

##### Evaluation

Once the values for blood Phe concentration, Phe tolerance and synthetic amino acid requirement have been calculated, a critical benefit analysis is performed, first within the team treating the patient and then together with the patient’s family. Unequivocal benefit from a medical perspective is considered as an increase in Phe tolerance by ≥100% and a 50% reduction in amino acid mixture intake. Minor changes must be individually evaluated in the overall clinical context. For patients with Phe values outside the target range, the therapeutic effect may also consist of a substantial reduction in the mean Phe concentration.

## Conclusions

European guidelines to optimize PKU care have been recently published [29] and sapropterin was approved to treat patients with BH_4_ responsive PAH deficiency who are younger than 4 years. This article provides practical recommendations for performing a BH_4_ loading test in newborns and discusses the possibility of initiating BH_4_ therapy from birth. The justification and benefits of this are summarized in Table 1. While the implementation of BH_4_ treatment is relatively similar in patients 0–4 years of age as it is in those > 4 years, the neonatal BH_4_ loading test allows early diagnosis and potential immediacy of treatment. Critical to this is the ready availability of sapropterin dihydrochloride tablets at the clinical center for neonatal screening, so that there is no delay between testing and treatment. Placing a patient on pharmacotherapy is time-consuming and requires diligence from both the family and the medical care team; therefore, for all patients there should be regular attempts to increase the dietary Phe intake, as this often succeeds without an increase in the blood Phe concentration. This article provides practical insights for performing a BH_4_ loading test in newborns and initiating BH_4_ therapy from birth; it also highlights a pharmacotherapeutic treatment option for some patients previously managed by diet alone.

## Additional files

Additional file 1: Dose dependent dissolution recommendation for sapropterin according to the guidance for water dilution. This table shows the number of tablets and the quantity of diluent required according to the weight of each child. (PDF 181 kb)

Additional file 2: Time course of capillary blood samples taken during the BH_4_ loading test. This scheme shows the timing of the blood samples during the BH_4_ loading test and guidance on how to interpret the results from the test. (JPG 40 kb)

## Abbreviations

ACMG: American College of Medical Genetics and Genomics
AS: quantity of protein from the amino-acid mixture
BH_4_: tetrahydrobiopterin
DHPR: dihydropteridine reductase
GMDI: Genetic Metabolic Dietician’s International
GTPCH: guanosine triphosphate cyclohydrolase
HPA: hyperphenylalaninemia
PAH: phenylalanine hydroxylase
Phe: phenylalanine
PKU: phenylketonuria
PTPS: 6-pyruvoyl-tetrahydropterin synthase
Tol: tolerance

## References

[1] Blau N, van Spronsen FJ, Levy HL (2010). Phenylketonuria. Lancet.

[2] Feillet F, van Spronsen FJ, MacDonald A, Trefz FK, Demirkol M, Giovannini M, Belanger-Quintana A, Blau N (2010). Challenges and pitfalls in the management of phenylketonuria. Pediatrics.

[3] Ozalp I, Coskun T, Tokatli A, Kalkanoglu HS, Dursun A, Tokol S, Koksal G, Ozguc M, Kose R (2001). Newborn PKU screening in Turkey: at present and organization for future. Turk J Pediatr.

[4] Guldberg P, Henriksen KF, Sipila I, Guttler F, de la Chapelle A (1995). Phenylketonuria in a low incidence population: molecular characterisation of mutations in Finland. J Med Genet.

[5] Aoki K, Ohwada M, Kitagawa T (2007). Long-term follow-up study of patients with phenylketonuria detected by the newborn screening programme in Japan. J Inherit Metab Dis.

[6] Vockley J, Andersson HC, Antshel KM, Braverman NE, Burton BK, Frazier DM, Mitchell J, Smith WE, Thompson BH, Berry SA (2014). Phenylalanine hydroxylase deficiency: diagnosis and management guideline. Genet Med.

[7] NSP (2004). Management of PKU.

[8] Loeber JG (2007). Neonatal screening in Europe; the situation in 2004. J Inherit Metab Dis.

[9] Zhou YA, Ma YX, Zhang QB, Gao WH, Liu JP, Yang JP, Zhang GX, Zhang XG, Yu L (2012). Mutations of the phenylalanine hydroxylase gene in patients with phenylketonuria in Shanxi, China. Genet Mol Biol.

[10] Mei L, Song P, Xu L (2013). Newborn screening and related policy against phenylketonuria in China. Intractable Rare Dis Res.

[11] Targum SD, Lang W (2010). Neurobehavioral problems associated with phenylketonuria. Psychiatry (Edgmont).

[12] Bernegger C, Blau N (2002). High frequency of tetrahydrobiopterin-responsiveness among hyperphenylalaninemias: a study of 1,919 patients observed from 1988 to 2002. Mol Genet Metab.

[13] Mitchell JJ, Wilcken B, Alexander I, Ellaway C, O'Grady H, Wiley V, Earl J, Christodoulou J (2005). Tetrahydrobiopterin-responsive phenylketonuria: the New South Wales experience. Mol Genet Metab.

[14] Muntau AC, Roschinger W, Habich M, Demmelmair H, Hoffmann B, Sommerhoff CP, Roscher AA (2002). Tetrahydrobiopterin as an alternative treatment for mild phenylketonuria. N Engl J Med.

[15] Kuvan SmPC [http://www.ema.europa.eu/docs/en_GB/document_library/EPAR_-_Product_Information/human/000943/WC500045038.pdf]. Accessed 17 Sept 2018.

[16] Fiori L, Fiege B, Riva E, Giovannini M (2005). Incidence of BH4-responsiveness in phenylalanine-hydroxylase-deficient Italian patients. Mol Genet Metab.

[17] Hennermann JB, Buhrer C, Blau N, Vetter B, Monch E (2005). Long-term treatment with tetrahydrobiopterin increases phenylalanine tolerance in children with severe phenotype of phenylketonuria. Mol Genet Metab.

[18] Keil S, Anjema K, van Spronsen FJ, Lambruschini N, Burlina A, Belanger-Quintana A, Couce ML, Feillet F, Cerone R, Lotz-Havla AS (2013). Long-term follow-up and outcome of phenylketonuria patients on sapropterin: a retrospective study. Pediatrics.

[19] Zimmermann M, Jacobs P, Fingerhut R, Torresani T, Thony B, Blau N, Baumgartner MR, Rohrbach M (2012). Positive effect of a simplified diet on blood phenylalanine control in different phenylketonuria variants, characterized by newborn BH4 loading test and PAH analysis. Mol Genet Metab.

[20] Leuret O, Barth M, Kuster A, Eyer D, de Parscau L, Odent S, Gilbert-Dussardier B, Feillet F, Labarthe F (2012). Efficacy and safety of BH4 before the age of 4 years in patients with mild phenylketonuria. J Inherit Metab Dis.

[21] Longo N, Siriwardena K, Feigenbaum A, Dimmock D, Burton BK, Stockler S, Waisbren S, Lang W, Jurecki E, Zhang C (2015). Long-term developmental progression in infants and young children taking sapropterin for phenylketonuria: a two-year analysis of safety and efficacy. Genet Med.

[22] Muntau AC, Burlina A, Eyskens F, Freisinger P, De Laet C, Leuzzi V, Rutsch F, Sivri HS, Vijay S, Bal MO (2017). Efficacy, safety and population pharmacokinetics of sapropterin in PKU patients <4 years: results from the SPARK open-label, multicentre, randomized phase IIIb trial. Orphanet J Rare Dis.

[23] Shintaku H, Ohura T (2014). Sapropterin is safe and effective in patients less than 4-years-old with BH4-responsive phenylalanine hydrolase deficiency. J Pediatr.

[24] Trefz FK, Scheible D, Frauendienst-Egger G (2010). Long-term follow-up of patients with phenylketonuria receiving tetrahydrobiopterin treatment. J Inherit Metab Dis.

[25] EPAR summary for the public: Kuvan (sapropterin dihydrochloride) [http://www.ema.europa.eu/docs/en_GB/document_library/EPAR_-_Summary_for_the_public/human/000943/WC500045034.pdf]. Accessed 17 Sept 2018.

[26] Blau N, Belanger-Quintana A, Demirkol M, Feillet F, Giovannini M, MacDonald A, Trefz FK, van Spronsen FJ (2009). Optimizing the use of sapropterin (BH(4)) in the management of phenylketonuria. Mol Genet Metab.

[27] Singh RH, Rohr F, Frazier D, Cunningham A, Mofidi S, Ogata B, Splett PL, Moseley K, Huntington K, Acosta PB (2014). Recommendations for the nutrition management of phenylalanine hydroxylase deficiency. Genet Med.

[28] Danecka MK, Woidy M, Zschocke J, Feillet F, Muntau AC, Gersting SW (2015). Mapping the functional landscape of frequent phenylalanine hydroxylase (PAH) genotypes promotes personalised medicine in phenylketonuria. J Med Genet.

[29] van Wegberg AMJ, MacDonald A, Ahring K, Belanger-Quintana A, Blau N, Bosch AM, Burlina A, Campistol J, Feillet F, Gizewska M (2017). The complete European guidelines on phenylketonuria: diagnosis and treatment. Orphanet J Rare Dis.

[30] Anjema K, Hofstede FC, Bosch AM, Rubio-Gozalbo ME, de Vries MC, Boelen CC, van Rijn M, van Spronsen FJ (2016). The neonatal tetrahydrobiopterin loading test in phenylketonuria: what is the predictive value?. Orphanet J Rare Dis.

[31] Longo N (2009). Disorders of biopterin metabolism. J Inherit Metab Dis.

[32] Staudigl M, Gersting SW, Danecka MK, Messing DD, Woidy M, Pinkas D, Kemter KF, Blau N, Muntau AC (2011). The interplay between genotype, metabolic state and cofactor treatment governs phenylalanine hydroxylase function and drug response. Hum Mol Genet.

[33] Musson DG, Kramer WG, Foehr ED, Bieberdorf FA, Hornfeldt CS, Kim SS, Dorenbaum A (2010). Relative bioavailability of sapropterin from intact and dissolved sapropterin dihydrochloride tablets and the effects of food: a randomized, open-label, crossover study in healthy adults. Clin Ther.

[34] Trefz FK, Burton BK, Longo N, Casanova MM, Gruskin DJ, Dorenbaum A, Kakkis ED, Crombez EA, Grange DK, Harmatz P (2009). Efficacy of sapropterin dihydrochloride in increasing phenylalanine tolerance in children with phenylketonuria: a phase III, randomized, double-blind, placebo-controlled study. J Pediatr.

