# Supplementary material for: Diagnostic and therapeutic recommendations for the treatment of hyperphenylalaninemia in patients 0–4 years of age
Source: Orphanet J Rare Dis. 2018 Sep 29;13:173. doi: 10.1186/s13023-018-0911-6 (PMC6162894; doi:10.1186/s13023-018-0911-6)
Supplement: Supplementary file 1 — Dose dependent dissolution recommendation for sapropterin according to the guidance for water dilution. This table shows the number of tablets and the quantity of diluent required according to the weight of each child. (PDF 181 kb) [file 13023_2018_911_MOESM1_ESM.pdf]

Dose-dependent dissolution recommendations for sapropterin according to the guidance for water dilution

10 mg/kg per day			Dose table for children weighing up to 20 kg	
Weight (kg)	Total dose (mg/day)	Number of tablets to be dissolved in water	Volume of water to be added (mL)	Volume of solution to be administered to the child (mL)
2	20	1	20	4
3	30	1	20	6
4	40	1	20	8
5	50	1	20	10
6	60	1	20	12
7	70	1	20	14
8	80	1	20	16
9	90	1	20	18
10	100	1	20	20
11	110	2	40	22
12	120	2	40	24
13	130	2	40	26
14	140	2	40	28
15	150	2	40	30
16	160	2	40	32
17	170	2	40	34
18	180	2	40	36
19	190	2	40	38
20	200	2	40	40
20 mg/kg per day			Dose table for children weighing up to 20 kg	
2	40	1	20	8
3	60	1	20	12
4	80	1	20	16
5	100	1	20	20
6	120	2	40	24
7	140	2	40	28
8	160	2	40	32
9	180	2	40	36
10	200	2	40	40
11	220	3	60	44
12	240	3	60	48
13	260	3	60	52
14	280	3	60	56
15	300	3	60	60
16	320	4	80	64
17	340	4	80	68
18	360	4	80	72
19	380	4	80	76
20	400	4	80	80
