# Supplementary figures and images for: Diagnostic and therapeutic recommendations for the treatment of hyperphenylalaninemia in patients 0–4 years of age
Source: Orphanet J Rare Dis. 2018 Sep 29;13:173. doi: 10.1186/s13023-018-0911-6 (PMC6162894; doi:10.1186/s13023-018-0911-6)

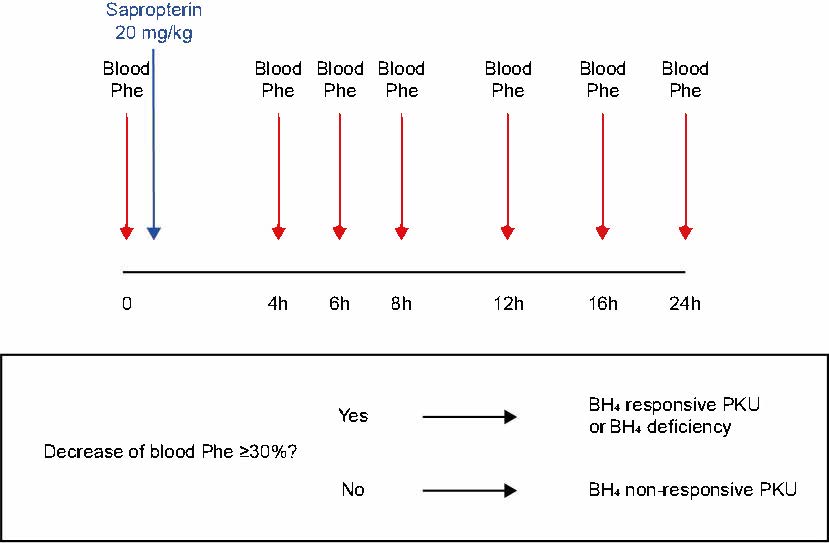

Supplement: Supplementary file 2 — Time course of capillary blood samples taken during the BH4 loading test. This scheme shows the timing of the blood samples during the BH4 loading test and guidance on how to interpret the results from the test. (JPG 40 kb) [file 13023_2018_911_MOESM2_ESM.jpg]
